# The ascorbic acid content of tomato fruits is associated with the expression of genes involved in pectin degradation

**DOI:** 10.1186/1471-2229-10-163

**Published:** 2010-08-06

**Authors:** Antonio Di Matteo, Adriana Sacco, Milena Anacleria, Mario Pezzotti, Massimo Delledonne, Alberto Ferrarini, Luigi Frusciante, Amalia Barone

**Affiliations:** 1 Department of Soil, Plant, Environment and Animal Sciences, University of Naples "Federico II", Via Università 100, 80055 Portici, Italy; 2 Department of Biotechnology, University of Verona, Strada Le Grazie 15 - 37134 Verona, Italy

## Abstract

**Background:**

High levels of ascorbic acid (AsA) in tomato fruits provide health benefits for humans and also play an important role in several aspects of plant life. Although AsA metabolism has been characterized in detail, the genetic mechanisms controlling AsA accumulation in tomatoes are poorly understood. The transcriptional control of AsA levels in fruits can be investigated by combining the advanced genetic and genomic resources currently available for tomato. A comparative transcriptomic analysis of fruit tissues was carried out on an introgression line containing a QTL promoting AsA accumulation in the fruit, using a parental cultivar with lower AsA levels as a reference.

**Results:**

Introgression line IL 12-4 (*S. pennellii *in a *S. lycopersicum *background) was selected for transcriptomic analysis because it maintained differences in AsA levels compared to the parental genotypes M82 and *S. pennellii *over three consecutive trials. Comparative microarray analysis of IL 12-4 and M82 fruits over a 2-year period allowed 253 differentially-expressed genes to be identified, suggesting that AsA accumulation in IL 12-4 may be caused by a combination of increased metabolic flux and reduced utilization of AsA. In particular, the upregulation of a pectinesterase and two polygalacturonases suggests that AsA accumulation in IL12-4 fruit is mainly achieved by increasing flux through the L-galactonic acid pathway, which is driven by pectin degradation and may be triggered by ethylene.

**Conclusions:**

Based on functional annotation, gene ontology classification and hierarchical clustering, a subset of the 253 differentially-expressed transcripts was used to develop a model to explain the higher AsA content in IL 12-4 fruits in terms of metabolic flux, precursor availability, demand for antioxidants, abundance of reactive oxygen species and ethylene signaling.

## Background

Oxidation reactions are essential for life, but they produce reactive oxygen species that can cause significant damage to cells. Therefore, complex protection systems have evolved based on antioxidants that help to eliminate these dangerous molecules [1]. Oxidative stress plays a role in many human diseases, but its impact can be reduced by the consumption of dietary antioxidants such as ascorbic acid (AsA), which is also known as vitamin C [2]. Humans and other primates are unable to synthesize AsA because the final step in its biosynthesis is blocked. Therefore, there has been great interest in the development of genetically modified food crops with high levels of antioxidants such as AsA [3,4]. As well as providing health benefits to humans, higher AsA levels improve both biotic and abiotic stress tolerance in plants [5,6] and enhance postharvest fruit quality [7]. The amount of AsA in plant cells depends on the strict regulation of its synthesis [8], metabolic recycling and degradation [9], and its transport [10]. The recycling of AsA is particularly important under stress conditions because reduced AsA is converted into an unstable radical (monodehydroascorbic acid), which dissociates into AsA and dehydroascorbic acid. Since the latter is also unstable and is rapidly degraded, the AsA pool can be depleted if the oxidized forms are not recovered by two reductases: monodehydroascorbic acid reductase (MDHAR) and dehydroascorbic acid reductase (DHAR) [11]. Both enzymes have been targeted by genetic engineering, their overexpression leading to elevated AsA levels [12] and, in the case of MDHAR, increased stress tolerance [13].

**Figure 1:**
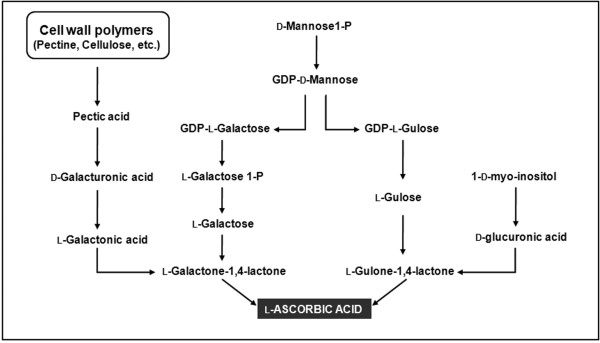
**Alternative pathways for l-Ascorbic acid biosynthesis in plants**. From left to right: d-galacturonate pathway [18], l-galactose pathway [11], l-gulose pathway [16] and *myo*-inositol pathway [17].

Although several metabolic pathways converge to generate AsA in plants [14] the l-galactose Wheeler-Smirnoff pathway is considered the primary route (Figure 1) and the roles of many of the genes and enzymes have been confirmed [15]. l-gulose [16] and myo-inositol have also been proposed as intermediates in AsA biosynthesis, indicating that part of the animal pathway could also operate in plants [17]. An alternative pathway with an l-galactonic acid intermediate has been also reported in strawberry [18] and grape fruit [19].

Although tomato fruits are considered a good dietary source of AsA, cultivated varieties of *Solanum lycopersicum *tend to have much lower levels than wild progenitors such as *S. pennellii *[20]. This reflects a range of genetic and environmental factors that result in quantitative variation across varieties and wild species [21]. The AsA content of tomato fruits is therefore suitable for QTL analysis [20,22]. Differences among several varieties have been described based on the metabolite content and antioxidant activities [23], but the precise genetic mechanisms controlling AsA levels remain elusive. Some insight has been gained by introgressing segments of the *S. pennellii *genome into a *S. lycopersicum *background [24] and identifying QTLs for fruit AsA content [20,22,25].

As tomato genomic resources have become more abundant [26], it has been possible to investigate the transcriptional control of fruit soluble solid content (Brix) by studying the transcriptomic changes in introgression lines with different Brix levels [27]. This type of analysis could also provide insight into the genetic mechanisms controlling AsA metabolic pathways. When this investigation began, 22,250 tomato Tentative Consensus sequences (TCs) were available in the TIGR database Gene Index Release 11.0 (June 21, 2006; http://compbio.dfci.harvard.edu/tgi/cgi-bin/tgi/gimain.pl?gudb=tomato). This facilitated the fabrication of the CombiMatrix TomatArray 1.0, a versatile tomato oligonucleotide microarray containing 20,200 specific 35-40 mers, each replicated four times [28]. Using this platform, we set out to determine whether the higher AsA levels in the fruits of an introgression line were associated with specific changes in steady state mRNA levels, which might provide some insight into the transcriptional regulation of AsA synthesis. Several genes with differential expression between the introgression line and the parental cultivated variety were identified, allowing us to develop a unified model that explains AsA synthesis in terms of the regulation of specific functional groups of genes.

## Results

### Ascorbic acid content

**Figure 2:**
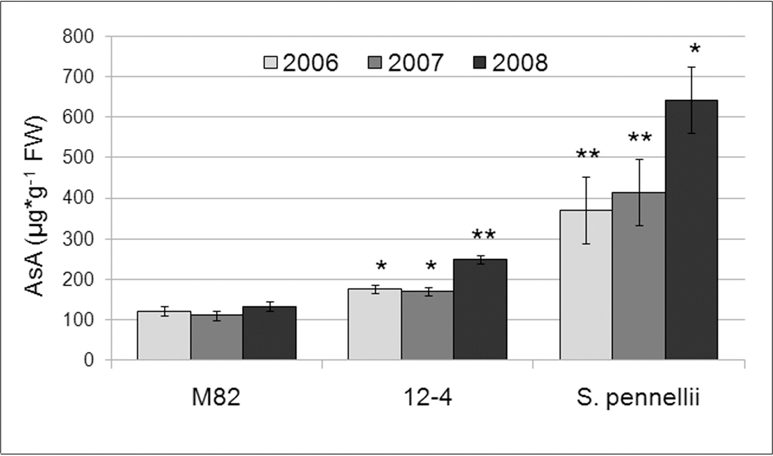
**Ascorbic acid (AsA) concentration in ripe fruit from the tomato IL 12-4 and its parental lines**. AsA concentration is expressed as μg * g^-1 ^fresh weight (FW). Mean values ± SE are reported for three consecutive greenhouse trials (in 2006, 2007 and 2008). An asterisk indicates that differences between IL 12-4 and *S. lycopersicum *cv. M82 are statistically significant in Student's t-test. *: 0.001 < P < 0.01; **: P < 0.001.

Within the framework of a research project to investigate QTLs controlling tomato fruit quality, we analyzed a number of introgression lines in which segments of the *S. pennellii *genome were introgressed into *S. lycopersicum cv*. M82. We selected IL12-4 for further analysis because it maintained differences in AsA levels compared to the parental genotypes M82 and *S. pennellii *over three consecutive trials (years 2006, 2007, 2008) in a greenhouse environment (Figure 2).

The average AsA concentrations in ripe M82 and *S. pennellii *fruit were 122 and 475 μg g^-1 ^fresh weight (FW), respectively, whereas in IL 12-4 the average AsA concentration was 199 μg g^-1 ^FW. These differences were statistically significant (Univariate ANOVA procedure; F_2, 41 _= 53.13; P < 0.001), and Dunnet's post-hoc test revealed significant differences when IL12-4 and *S. pennellii *were separately compared with M82 (P < 0.05). A significant genotype × year interaction over the three consecutive trials was also observed (Univariate ANOVA model, F_4,41 _= 3.060, P < 0.05). Our analysis therefore confirmed that the introgression of *S. pennellii *genetic material into IL12-4 contributed to the higher fruit AsA content compared to M82. Indeed, the AsA content in IL12-4 fruit was on average 45% higher than M82 in 2006, 55% higher in 2007 and 87% higher in 2008. Therefore, IL12-4 was chosen for further comparative transcriptome analysis together with the parental line M82.

### Comparative microarray analysis

In order to identify transcripts expressed at different levels in the two selected genotypes, total RNA was extracted from three biological replicate samples taken from each genotype in two consecutive years (2007 and 2008). Differential transcript accumulation was documented using single-color hybridization on the TomatArray 1.0 microarray followed by a two-factorial ANOVA test (P < 0.01) with the M82 transcriptome as a reference. Because small changes in gene expression might underlie differences in AsA accumulation, differentially-expressed transcripts were not filtered using a fold-change threshold, and differences were considered irrespective of the intensity of the change. Thus we identified 253 sequences (1.17% of those represented on the TomatArray1.0) that were differentially expressed at the red-ripe stage (Additional file 1), 7.9% of which significantly matched (e value <1 × 10^-10^) with non-annotated sequences in the NCBI's non-redundant NR database and 7.5% of which showed no matches. Among these transcripts, 61 (24.1%) were upregulated and 192 (75.9%) were downregulated in IL 12-4.

The distribution of GO categories according to Biological Process (BP), Molecular Function (MF) and Cellular Component (CC) was complex. The BP terms "transport", "cellular component organization and biogenesis" and "amino acid and derivative metabolic process" were the highest ranked (14.8%, 11.8% and 10.1%, respectively) and were similarly represented among both the upregulated and downregulated genes (Additional file 2). The MF terms "hydrolase activity", "protein binding" and "nucleotide binding" occurred most frequently (21.1%, 18.4% and 14.7%, respectively) and again were similarly represented among the upregulated and downregulated genes (Additional file 3). Finally, the most frequent CC terms indicated that the differentially expressed genes were preferentially active in the "plastid" (33.9%), "mitochondrion" (22.6%) and "ribosome" (8.7%) (Additional file 4). Notably, this preferential order was preserved for downregulated genes but not for upregulated genes, perhaps because of the paucity of the latter group (61 sequences and 25 classifications).

Hierarchical clustering (HC) using Pearson's correlation metric identified sub-clusters of tightly co-regulated transcripts, which should provide new insights in to the transcriptional regulation of AsA metabolism and provide putative functions for non-annotated sequences. Among the transcripts that are upregulated in IL 12-4 (Additional file 5) the HC output indicated a sub-cluster that grouped together sequences such as a propionyl carboxylase beta chain (TC177185), two pyridoxal-phosphate dependent TrpB-like enzymes (TC172849 and TC183991), a pectinesterase family protein (TC177576), a 21-kDa protein precursor (TC182308) and a number of non-annotated transcripts. Another cluster brought together ethylene-related transcripts such as a 1-aminocyclopropane-1-carboxylate synthase (TC169916) and a cystathionine γ-synthase (TC184006), with an amino acid permease-like protein (TC170812), a DnaJ-like chaperone protein (TC174366), a phosphoglycerate kinase (TC190107) and again a number of non-annotated transcripts. In contrast, among the transcripts that are downregulated in IL 12-4 (Additional file 6), an AsA peroxidase (TC172881) was coregulated with a Sec23-Sec24 transport family protein (TC188915), a β-glucosidase (TC170324), a Dof zinc finger protein (TC188776), a SelT-like protein precursor (TC174342) and a small number of non-annotated transcripts.

Based on functional annotation, gene ontology (GO) classification and hierarchical clustering, a subset of the 253 differentially-expressed TCs (Table 1) was used to develop a model that could explain the higher AsA content in IL 12-4. Within this select group, four TCs were related to AsA pathways, six to hormone metabolism, five to glycolysis and the Calvin cycle and three to glutathione metabolism. Together, these help to characterize the essential changes in IL 12-4 cellular metabolism that may underpin the increased accumulation of AsA in the fruits. A further six TCs involved in stress responses and three involved in plastid metabolism could also be linked into the model.

**Table 1:** List of TCs affecting AsA accumulation that are differentially expressed in IL 12-4 and M82 according to microarray data.

TC ID	Fold change IL12-4 *vs*. M82	Annotation
**AsA pathways**		
TC177576	4.439	pectinesterase family protein
TC182248	-1.198	beta-glucuronidase precursor
TC170324	-1.370	beta-glucosidase 01
TC172881	-1.360	ascorbate peroxidase
**Hormone metabolism**		
TC172849	4.864	pyridoxal-phosphate dependent-like enzyme
TC183991	3.812	pyridoxal-phosphate dependent-like enzyme
TC184220	2.611	adenosine 5'-phosphosulfate reductase
TC184006	2.504	cystathionine gamma-synthase
TC169916	1.443	1-aminocyclopropane-1-carboxylate synthase
TC172320	-0.901	s-adenosylmethionine-dependent methyltransferase
**Glycolysis and Calvin cycle**		
TC188751	4.842	RuBisCO small subunit protein
TC190107	1.868	phospho-glycerate kinase
TC182193	1.539	RuBisCO subunit binding-protein alpha subunit
TC183220	0.961	pfkb-type carbohydrate kinase family protein
TC172505	-1.166	pyruvate kinase
**Glutathione metabolism**		
TC175970	-0.542	spermidine synthase
TC181406	-1.095	cysteine synthase
TC189778	-9.031	glutathione s-transferase
**Stress response**		
TC180786	4.167	wound responsive protein
TC174575	1.531	anther-specific proline-rich apg-like protein
TC170015	-3.196	stress-related protein
TC182497	-3.783	calmodulin
TC180230	-4.273	pto kinase interactor 1
TC180552	-4.567	leucine-rich repeat receptor-like kinase
**Plastid metabolism**		
TC185020	1.011	NADP adrenodoxin-like ferredoxin reductase
TC186521	-1.214	ATPase-like protein
TC180781	-2.197	ATP synthase subunit h family protein

### Expression of transcripts related to AsA metabolism

**Figure 3:**
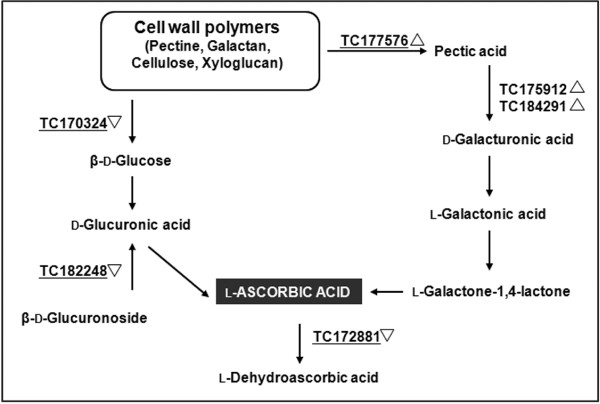
**Network showing the differential expression (between IL 12-4 and M82) of genes associated with AsA metabolism in fruit**. Underlined TCs are differentially expressed in the microarray experiment, with upright triangles representing upregulation and inverted triangles representing downregulation.

Among the different AsA synthesis pathways mentioned above, our microarray data ruled out the Wheeler-Smirnoff pathway as the predominant source of higher AsA levels in IL 12-4 fruit because probes specific for genes controlling key steps in this pathway (e.g. genes encoding GDP-d-mannose pyrophosphorylase, GDP-l-galactose phosphorylase, l-galactose-1-P phosphatase and l-galactose dehydrogenase) were included on the chip and showed no evidence of differential expression. However, among 23 probes representing putative pectinesterase transcripts, one in particular (TC177576) was 4.4-fold more abundant in IL12-4 than M82, suggesting that increased pectin degradation could provide much of the additional AsA in IL 12-4 fruits (Figure 3). The breakdown of other structural polymers in the cell wall did not seem to provide intermediates for AsA synthesis, e.g. transcripts for β-glucosidase (TC170324) and β-glucuronidase (TC182248) were repressed, which would delay the conversion of cellobiose into β-D-glucose and reduce the rate of D-glucuronic acid synthesis from β-D-glucuronoside. Finally, a putative AsA peroxidase transcript (TC172881) was downregulated (1.36-fold more abundant in M82 than IL12-4), suggesting the accumulation of AsA might also reflect a lower rate of oxidation.

**Figure 4:**
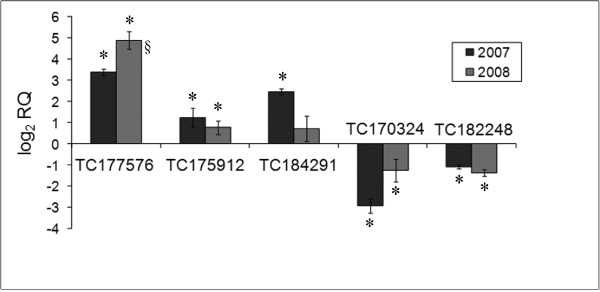
**Validation of differential expression by qRT-PCR**. Relative quantification (RQ) of transcripts associated with differential AsA accumulation in IL12-4 and M82 in years 2007 and 2008. Mean values ± SE are shown. An asterisk indicates that differences between IL 12-4 and *S. lycopersicum *cv. M82 are statistically significant in Student's t test (P < 0.05), and § indicates significant differences in expression between 2007 and 2008 (P < 0.05).

Upregulation of pectinesterase transcript TC177576 was confirmed in both the 2007 and 2008 samples by qRT-PCR (Figure 4). To investigate the possibility that pectin catabolism might boost AsA levels, perhaps by increasing flux through the L-galactonic acid pathway, we turned our attention to the transcripts for two putative polygalacturonases (TC175912 and TC184291), which had been overlooked in the original microarray experiment because they were filtered out of the raw data. The qRT-PCR results indicated that both were upregulated in IL12-4 compared to M82 (Figure 4). The downregulation of two TCs involved in the D-glucuronic acid pathway (TC170324 and TC182248) was also validated by qRT-PCR. A correlation analysis between microarray and qRT-PCR expression data (log_2 _of the expression value) yielded a Pearson's value of 0.878 (P < 0.001).

### Expression of transcripts related to ethylene metabolism

**Figure 5:**
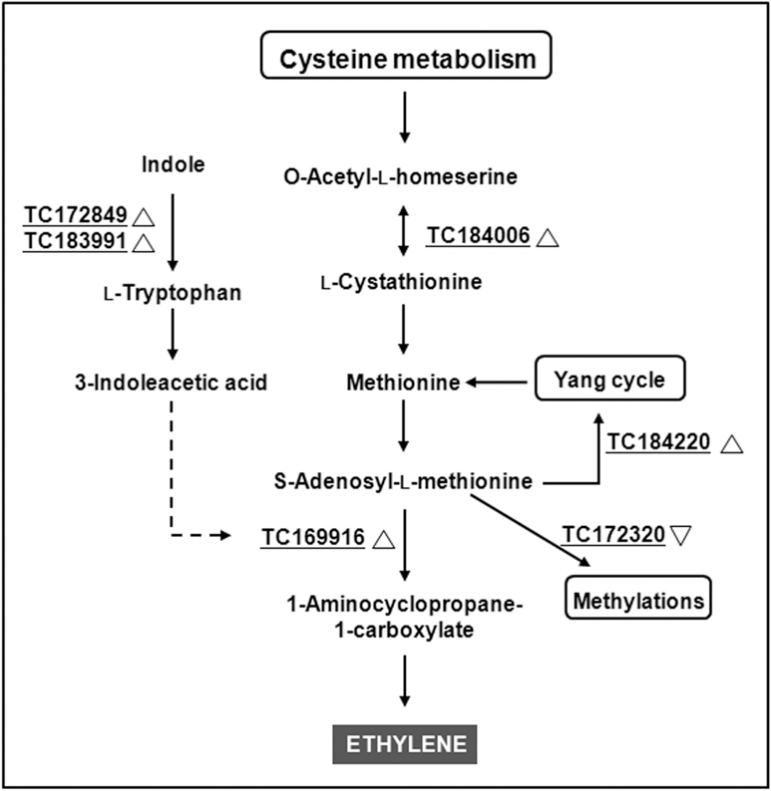
**Network showing the differential expression (between IL 12-4 and M82) of genes associated with ethylene metabolism**. Underlined TCs are differentially expressed in the microarray experiment, with upright triangles representing upregulation and inverted triangles representing downregulation. Dashed line indicates an external supply that can enhance enzyme activity.

Several transcripts possibly involved in ethylene biosynthesis were upregulated (Figure 5), including a putative cystathionine γ-synthase (TC184006). The TomatArray 1.0 included probes for six 1-aminocyclopropane-1-carboxylate (ACC) synthases and four ACC oxidases, providing a good representation of the key steps in ethylene biosynthesis. Among these, only one ACC synthase (TC169916) was upregulated. Another upregulated transcript (TC184220) that was not annotated automatically was manually identified as a putative adenosine 5'-phosphosulfate reductase, which is also involved in ethylene synthesis. In contrast, an S-adenosylmethionine-dependent methyltransferase (TC172320) was downregulated, which could reflect the reduced utilization of S-adenosylmethionine (AdoMet) for methylation and its diversion to ethylene biosynthesis. Two pyridoxal phosphate-dependent TrpB-like transcripts (TC172849 and TC183991) were also upregulated (29.12-fold and 14.04-fold higher in IL12-4, respectively). These are likely to be involved in tryptophan synthesis and thus may have an impact on the supply of 3-indoleacetic acid (IAA) with positive effects on ACC synthase activity and hence ethylene biosynthesis.

### Expression of transcripts related to glutathione metabolism

**Figure 6:**
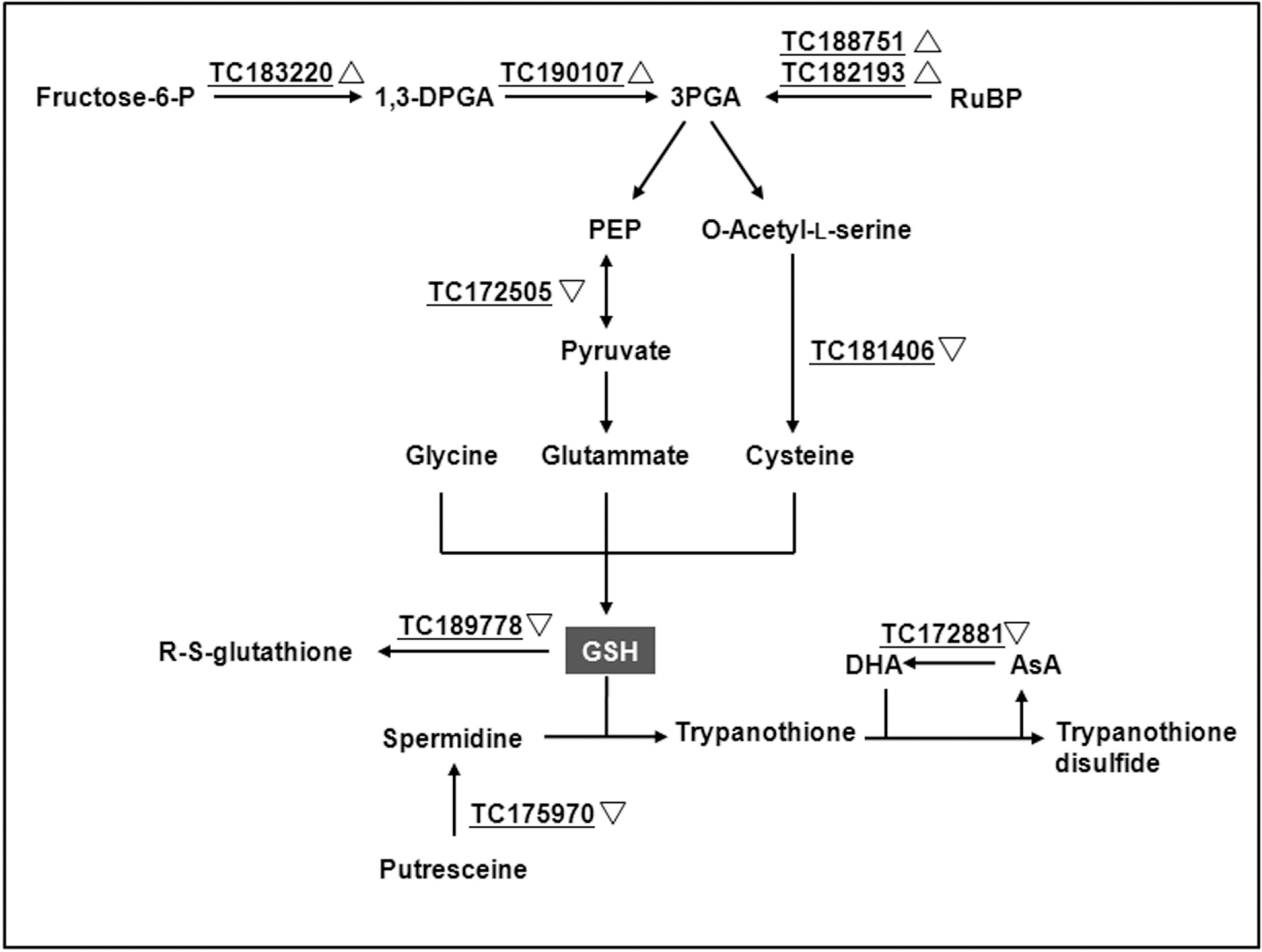
**Network showing the differential expression (between IL 12-4 and M82) of genes associated with the glycolytic pathway and glutathione metabolism**. Underlined TCs are differentially expressed in the microarray experiment, with upright triangles representing upregulation and inverted triangles representing downregulation. Abbreviations: 1,3-DPGA, 1,3-diphosphoglyceric acid; 3PGA, 3 phosphoglyceric acid; RuBP, Ribulose 1,5-biphosphate; PEP, phosphoenolpyruvic acid; GSH, glutathione; DHA, dehydroascorbic acid.

Several transcripts encoding enzymes in the glycolytic pathway were upregulated (Figure 6), including phosphoglycerate kinase (TC190107) and a phosphofructokinase b-type carbohydrate kinase (TC183220). Two transcripts encoding components of the RuBisCo complex were also upregulated (TC188751 and TC182193), suggesting a gene expression pattern converging on the accumulation of 3-phosphoglycerate (3-PGA). Downregulated transcripts included a pyruvate kinase (TC172505) and cysteine synthase (TC181406), possibly reflecting a lower flux in gluthathione biosynthesis. Consistent with this hypothesis, transcript TC189778 putatively encoding a gluthathione S-transferase was also downregulated. A transcript encoding spermidine synthase (TC175970) was also repressed, which is relevant because spermidine is a precursor of trypanothione, which spontaneously reduces dehydroascorbic acid to AsA. This fits well with the coordinated downregulation of AsA peroxidase (TC172881) described above.

## Discussion

The antioxidant activity of AsA makes it a crucial component of the stress response in plants [29]. However, the analysis of mutants deficient in AsA synthesis reveals downstream effects on hundreds of genes, suggesting it is also a key signaling molecule in defense and development [30]. To fulfill such an important role, AsA synthesis must be tightly regulated. Several alternative biosynthetic pathways have been identified and it is therefore difficult to pin down exactly how synthesis is controlled in the context of development, stress responses and normal homeostasis [14].

In order to determine how AsA synthesis is controlled in tomato fruits we combined introgression lines previously used for QTL mapping, and transcriptome analysis using the new CombiMatrix microarray platform. Because the AsA content of tomato fruits shows quantitative variation, introgression lines involving tomato varieties with strikingly different AsA levels are a useful tool to track down QTLs. Such lines have been generated by introgressing DNA from *S. pennellii*, a wild tomato species with high AsA levels, into the background of a common processing cultivar of *S. lycopersicum *(M82), which has a lower AsA content [20,22]. Among six QTLs affecting AsA levels that were identified by Rousseaux *et al*. [22], only one (asa12-4) increased fruit AsA concentration, and this was found in IL 12-4. Stevens *et al*. [20] also identified 11 QTLs affecting fruit AsA levels; eight increased the AsA content but none of them was observed in IL 12-4.

Under our experimental conditions, *S. pennellii *LA0716 achieved on average a 3.9-fold increase in fruit AsA levels compared to M82, showing that this wild species is a useful reservoir of genetic variability for tomato fruit quality enhancement. Our data confirmed those of Rosseaux *et al*. [22] who observed higher levels of AsA in IL12-4 fruits, but only in one season. The average AsA content in IL12-4 fruit is higher than that usually observed in commercial varieties [23,31,32], although there are some exceptions [33]. The asa12-4 QTL therefore provided us with a valuable tool to facilitate the transcriptomic analysis of fruit AsA metabolism in tomato even though asa12-4 does not increase AsA levels to those seen in *S. pennellii*.

Comparative microarray analysis revealed 253 differentially-expressed transcripts, 24% of which were upregulated in IL12-4 fruit and 76% of which were downregulated. Gene Ontology classifications automatically retrieved from the GO Consortium [34] using Blast2GO provided a useful framework for annotation, classification and comparison of groups of sequences according to biological process, molecular function and cellular component terms. Combining functional annotation and GO classifications with hierarchical clustering helped to identify groups of co-regulated transcripts. One of these (Additional file 5) included upregulated transcripts involved in glycolysis, the Calvin cycle, pectin breakdown, tryptophan synthesis and the wound response, whereas another (Additional file 6) included downregulated transcripts involved in AsA peroxidation, glucuronoside metabolism and glutathione metabolism.

Overall, our results suggested that metabolic changes associated with AsA accumulation in tomato fruits were focused on the L-galactonic acid and d-glucuronic acid pathways (Figure 3), rather than the main Smirnoff-Wheeler AsA pathway. Indeed, the upregulation of transcripts encoding a pectinesterase and two polygalacturonases suggests that the accelerated breakdown of pectin polymers may increase the availability of intermediates for the L-galactonic acid pathway, increasing the flux towards of AsA biosynthesis. The importance of the L-galactonic acid pathway in AsA biosynthesis was demonstrated in transgenic Arabidopsis plants overexpressing a strawberry D-galacturonic acid reductase; the transgenic plants had higher AsA levels than wild type plants, reflecting the conversion of D-galacturonic acid into L-galactonic acid as the key control point in the L-galactonic acid branch of AsA metabolism [18].

The above results suggest that accelerated pectin polymer breakdown increases the availability of precursors for AsA biosynthesis in the L-galactonic acid pathway, resulting in the accumulation of AsA in fruits. The breakdown of other cell wall polymers such as cellulose could have a similar impact, but our data strongly indicate that cellulose does not contribute to AsA biosynthesis in this manner. A β-glucosidase transcript is downregulated, indicating that precursors for the D-glucuronic acid branch of the pathway would become scarcer and flux would be reduced. The coordinate downregulation of a β-glucuronidase transcript also supports the hypothesis of reduced flux through the D-glucuronic acid pathway (Figure 3). Consistent with the hypothesis that substrate availability limits the amount of AsA produced via an alternative pathway, plants with lower pectate lyase activity accumulate less AsA [18].

Enhancing the breakdown of cell wall components therefore appears to be a useful strategy for the creation of transgenic fruit crops with higher levels of AsA. However, where this has been attempted, it has often led to changes in the process of fruit softening and overall firmness. The knockdown of polygalacturonase genes delays softening and thus increases shelf life [35] while making the fruits firmer [36]. In contrast, the knockdown of a pectin methylesterase had the opposite effect, significantly reducing shelf life [37]. This is in line with the reduced firmness of IL12-4 fruit we observed at the red-ripe stage (unpublished data), which provides further support for pectin catabolism as the main mechanism underlying the increase in AsA levels.

The higher accumulation of AsA in IL12-4 fruits may involve additional mechanisms that have not been elucidated so clearly as the pectin degradation process. For example, the upregulation of both a pyrophosphate-dependent phosphofructo-1-kinase and a phosphoglycerate kinase may indicate an increase in the catabolism of reducing monosaccharides in the cytosol. Nevertheless, glycolytic flux in IL12-4 fruit might be limited given the downregulation of a pyruvate kinase. Together with the upregulation of two RuBisCO components, the supply of 3-phosphoglycerate may increase (Figure 6), leading to an increase in the synthesis of hexoses that are then made available for the AsA biosynthetic pathway.

The higher AsA content in IL 12-4 fruits could also result from the reduced utilization of AsA, as indicated by the downregulation of an AsA peroxidase (Figure 3). This is also in line with the downregulation of a spermidine synthase (possibly involved in trypanothione biosynthesis), which would therefore be less required for dehydroascorbate regeneration to AsA (Figure 6). Accordingly, the downregulation of a pyruvate kinase and a cysteine synthase indicates that the synthesis of glutathione is repressed, while the downregulation of a gluthathione S-transferase indicates that glutathione utilization is also reduced. Taken together, these results are consistent with a hypothesis based on lower overall demand for cellular antioxidant activity, in turn reducing glutathione biosynthesis and allowing AsA to accumulate. Additional changes to plastid metabolism are evident from the data presented in Table 1, including the upregulation of two RuBisCO proteins and an NADP ferredoxin reductase, and the downregulation of an ATP synthase, all of which may be involved in reducing the abundance of reactive oxygen species by limiting the electron flux from water to NADPH [38,39]. The presence of fewer reactive oxygen species is also consistent with the observed downregulation of stress response genes encoding calmodulin, a Pto kinase-binding protein and a leucine-rich repeat receptor-like kinase.

Finally, given that pectin catabolism can be triggered by ethylene, we focused on the modulation of transcripts associated with the ethylene biosynthesis pathway. Some of the observed changes may account for major effects on ethylene biosynthetic flux, e.g. the upregulation of a cystathionine γ-synthase, which catalyzes the first committed step in methionine biosynthesis and whose role as a key step in the regulation of ethylene metabolism has been proven in transgenic tobacco plants overexpressing a truncated cystathionine γ-synthase gene [40]. Furthermore, the upregulation of a putative adenosine 5'-phosphosulfate transcript suggested that the efficiency of the Yang cycle may be increased [41] (Figure 5), and the downregulation of a putative S-adenosylmethionine-dependent methyltransferase suggested that AdoMet is being used less frequently for substrate methylations. Both these changes correspond to an increase in ethylene flux, as does the upregulation of a 1-aminocyclopropane-1-carboxylate (ACC) synthase. Indeed, ethylene production is tightly regulated by feedback control of ACC synthase and/or ACC oxidase (reviewed by Kende [42]). In tomato fruit, both ACC synthase and ACC oxidase activity are induced by exogenous ethylene [43], and the induction is achieved by transcriptional regulation [44]. ACC synthase may also promote ethylene biosynthesis driven by IAA [45], which is consistent with our observation that two tryptophan synthase transcripts are upregulated (Figure 5). Overall, the transcriptional modulation of genes associated with ethylene biosynthesis appears to enhance ethylene levels in IL 12-4 fruit, which may trigger the pectin degradation that leads to AsA accumulation.

## Conclusions

Comparative transcriptome analysis in tomato fruits from an introgression line with high levels of AsA and a parent with low levels has suggested that the higher level of AsA in IL12-4 fruits may reflect a combination of increased AsA synthesis and reduced utilization. In particular, the higher AsA content in IL 12-4 may be transcriptionally controlled through the upregulation of genes driving pectin degradation, thus releasing intermediates for the L-galactonic acid pathway, which is therefore likely to affect AsA biosynthesis in IL12-4 fruit. We found no evidence supporting the specific involvement of the glucuronic acid and Smirnoff-Wheeler pathways. The accelerated pectin degradation might itself be triggered by an increase in ethylene biosynthesis, which may be related to the upregulation of several genes in the ethylene biosynthetic pathway. An increase in the supply of hexoses for AsA biosynthesis may also help to boost AsA levels, which is suggested by the transcriptional upregulation of genes involved in processes converging on 3-PGA accumulation. Finally, we note that several plastidial genes identified in the comparative analysis could conceivably help to reduce the abundance of reactive oxygen species, in turn preventing AsA peroxidation and allowing AsA to accumulate.

It will be necessary to carry out additional metabolic studies to characterize the regulatory mechanisms we have identified, and to establish the chain of events leading to AsA accumulation and the trigger that induces it. We also need to determine whether AsA levels can be controlled through the L-galactonic acid pathway in tomato, as this has been demonstrated only in strawberry thus far, and whether this is a specific mechanism acting only in IL 12-4 fruit, or if it is a general mechanism controlling AsA accumulation in the fruits of all tomato varieties.

## Methods

### Plant material

The tomato plants used in this study were the introgression line (IL) 12-4 and its parental genotypes. IL 12-4 (accession LA4102) contains a 52 cM homozygous introgression from *S. pennellii *(accession LA0716) in a *S. lycopersicum cv*. M82 background (accession LA3475) [24]. LA0716 is a homozygous, self-fertile indeterminate accession from Atico, Peru, with green fruits. M82 is a determinate, red-fruited variety used for processing. All seeds were provided by the C.M. Rick Tomato Genetics Resource Center at the University of California (Davis, USA).

### Greenhouse trials

IL12-4 and its parental genotypes were cultivated over three consecutive years (2006-2008) in a greenhouse at the Department of Soil, Plant, Environmental and Animal Production Sciences at the University of Naples (Portici, Italy). Six plants from IL 12-4 and 15 from each parental line were transferred into 20-cm pots containing a 1:1 mixture of medium sandy soil and compost at the beginning of March. Pots were distributed randomly 15 cm apart in rows separated by a 50-cm channel, and were supplemented with Nitrophoska Blu Spezial 12-12-17 (+2+20) (Compu) slow-release fertilizer (5 g). Plants were watered twice daily using an automated irrigation device with individual drip lines. Prior to flowering, the plants were supplied every two weeks with 30-10-10 liquid fertilizer (Grow More, USA). Fruits were collected from IL 12-4 and its cultivated parent when 75% were full sized and red-ripe, softening had increased and the inside of the columella was completely red. For *S. pennellii*, the maturity of the green fruit was based on size and softness. Samples were generated by pooling ripe fruits from the same plant (at least three samples per line) and discarding the seeds, jelly parenchyma, columella and placenta tissues. Samples were frozen under liquid nitrogen and stored at -80°C prior to homogenization in a Waring blender and processing for the extraction of total RNA and AsA.

### Ascorbic acid quantification

AsA levels were measured using a modified version of the procedure described by Kampfenkel *et al*. [46]. Frozen tissue (250 mg) was placed in a 1.5-ml tube with a bead and 200 μl of ice-cold 6% trichloroacetic acid (TCA) (Sigma), and was homogenized at 50 Hz in a TissueLyzer (Qiagen) for 2 × 1 min. Samples were then incubated on ice for 10 min and centrifuged for 25 min at 25,000 × *g *and 4°C. The supernatant was supplemented with 6% TCA to a total volume of 500 μl, and then centrifuged as above for 10 min. A 50-μl aliquot was transferred to a fresh 1.5-ml tube containing 150 μl 0.2 M phosphate buffer (pH 7.4) and this was supplemented with 50 μl double distilled water, 250 μl 10% TCA, 200 μl 42% H_3_PO_4_, 200 μl 2,2′-dipyridyl and 100 μl 3% FeCl_3_. The mixtures were vortexed and incubated at 42°C for 40 min prior to measurement at 525 nm in a Beckman DU-640 UV spectrophotometer using 6% TCA as a reference. The AsA concentration was expressed in μmol g^-1 ^fresh weight according to the standard curve A_525 _= 3.6593 × μmol AsA, designed over a dynamic range of 0-0.7 μmol AsA (R^2 ^= 0.9982). The value was then converted into μg g^-1^.

### Statistical analysis of phenotypic data

Statistical analysis was performed using SPSS 15.0 for Windows (evaluation version release 15.0.0). The significance of genotype with respect to AsA levels in fruit over three consecutive greenhouse trials was determined by comparing mean AsA levels in IL 12-4, M82 and *S. pennellii *samples using a Univariate ANOVA with Dunnet's post-hoc test. Because of the significant interaction between genotype and year (P < 0.05), an independent-sample Student's t-test was used to compare IL12-4 and its wild parent to the M82 reference within each trial.

### Chip design and synthesis

Transcriptomic analysis was performed on a 90K TomatArray1.0 microarray synthesized using the CombiMatrix platform at the Plant Functional Genomics Center of the University of Verona http://ddlab.sci.univr.it/FunctionalGenomics/. CombiMatrix technology combines phosphoramidite chemistry and semiconductors for the digital control of probe synthesis on the chip surface. The 90K TomatArray1.0 contains 90,000 siliceous electrodes (features) supporting 20,200 unique 35-40 mer DNA oligonucleotide probes synthesized *in situ *with four replications. Probes were designed to target single transcripts using OligoArray 2.1 [47] to match specifically with the 21,550 tomato Tentative Consensus sequences (TCs) available in the TIGR database Gene Index Release 11.0 (June 21, 2006). Missed TCs were not represented on the chip because it was not possible to design specific probes with the necessary thermodynamics. Nine bacterial oligonucleotide sequences provided by CombiMatrix were used as negative controls. The four replicates of each probe were distributed randomly across the array to control for internal variability.

### RNA isolation and microarray hybridization

Total RNA was extracted from frozen, homogenized and powdered tomato fruit tissue using the CTAB (hexadecyltrimethylammonium bromide) method [48]. Samples were taken from IL12-4 and M82 using three plants per genotype in the 2007 and 2008 growing trials.

Microarray experiments were designed and conducted according to the MIAME guidelines http://www.mged.org/miame. Total RNA (1 μg) was used as a template to synthesize antisense RNA (aRNA) with the SuperScript™ Indirect RNA Amplification System Kit (Invitrogen) incorporating Alexa Fluor 647 Reactive Dye. Pre-hybridization, RNA fragmentation, hybridization with 3 μg of labeled and fragmented aRNA and post-hybridization washes were performed according to CombiMatrix protocols http://www.combimatrix.com/docs/PTL020_00_90K_Hyb_Imaging.pdf.

After hybridization and washing, the microarray was dipped in imaging solution, covered with LifterSlip™, and then scanned using a Perkin Elmer ScanArray 4000XL and the accompanying acquisition software (ScanArray Express Microarray Analysis System v4.0). The resulting TIFF images were processed to extract raw data using the CombiMatrix Microarray Imager Software v5.8.0. Signal probe medians and standard deviations were imported into the SPSS software, and normalization was achieved by correcting each probe median based on the ratio between the median of the array and the average median of arrays. Following data normalization and quality control, all values were log transformed (base 2). Finally, probe signals with a variability coefficient higher than 0.5 as well as spikes and factory probes were filtered out. Also, probes with signal intensities in the uppermost and lowermost 10% of values were deleted. The microarray data were deposited in Gene Expression Omnibus (GEO) under the series accession GSE19897.

Differential signals in the IL12-4 *vs*. M82 fruit transcriptomes were identified using the two-factor ANOVA module in the TIGR Multiple Experiment Viewer Software v4.0 http://www.tigr.org/software/tm4/[49]. Hierarchical clustering of differentially-expressed signals was achieved using Pearson correlation as a metric to investigate gene expression co-regulation.

Blast2GO http://blast2go.bioinfo.cipf.es/ was used to provide automatic high-throughput annotation, gene ontology mapping and categorization of TCs showing differential transcription signals [50]. Sequences whose annotation was not automatically provided through similarity matching in the NCBI's non-redundant NR database were processed manually using the similarity search tools FASTA33 http://www.ebi.ac.uk/Tools/fasta33/index.html and/or SGN BLAST http://sgn.cornell.edu/tools/blast/. In each case, an expectation value threshold of 10^-10 ^was used.

### Experimental validation

The expression profiles of TCs considered to be key control points for AsA accumulation were validated by real-time quantitative RT-PCR in a 7900HT Fast Real-Time PCR System (Applied Biosystems). Amplification was performed in 12.5-μl reaction volumes using a *Power *SYBR^® ^Green PCR Master Mix (Applied Biosystems). Relative quantification was achieved by the ΔΔCt method [51]. Primer pairs were validated using a standard curve over a dilution range 1-10^-3 ^(R^2 ^> 0.98; slope close to -3.32). The primer pair sequences are listed in Table 2.

**Table 2:** Primer pairs used for qPCR validation of genes involved in AsA accumulation that are differentially expressed in IL 12-4 and M82

	Forward Primer	Reverse Primer
TC170324	5'-aatcggtaactctggcactga-3'	3'-cagcagcatgagcaagaagt-5'
TC175912	5'-ccattcaagtcagccctttt-3'	3'-ggtagagcatgcaccagtttt-5'
TC177576	5'-taacatttgctgaggaaagatgca-3'	3'-tttttgaagtgtttgatcccattc-5'
TC182248	5'-ttcagctgtagcatgggttg-3'	3'-aaacaagattgcgaccactgt-5'
TC184291	5'-ggagagcagcatgtcaatca-3'	3'-ggcatttccttgtccgttta-5'

## Authors' contributions

ADM conceived the experiment, carried out plant growth and AsA quantification, microarray analysis, data mining and drafted the manuscript. AS contributed to plant growth and AsA quantification, and to the manuscript writing. MA carried out qRT-PCR experiments. AF carried out the chip synthesis and contributed to the microarray hybridization. MP participated to experimental design and to the manuscript revision. MD developed chip design and revised the manuscript. LF critically revised the manuscript. AB coordinated the study, partecipated to interpretation of data and largely contributed to the manuscript revision. All authors have read and approved the final manuscript.

## Supplementary Material

Additional file 1 **List of 253 probes showing differential hybridization signals in the IL12-4 and M82 transcriptomes**. Statistical and annotation details of 253 probes showing differential hybridization signals at a 2-Factorial ANOVA model (P < 0.01) in the IL12-4 transcriptome compared to M82. Statistical analysis was performed by the TM4:MeV microarray software suite and the IL 12-4 vs. M82 signal ratio together with the Adjusted P value were reported. The automatic BLAST annotation of TC sequences was performed by the BLAST2GO software suite and the expectation value (e value), sequence similarity and corresponding Gene Ontology terms were reported. TCs are listed according to a decreasing IL12-4 vs. M82 fold change. Click here for file

Additional file 2 **Ontology categorization for Biological Process**. Functional categorization according to GO Biological Process (BP) vocabulary of Tentative Consensus (TCs) showing differential hybridization signals in IL12-4 vs. M82. Click here for file

Additional file 3 **Ontology categorization for Molecular Function**. Functional categorization according to GO Molecular Function (MF) vocabulary of Tentative Consensus (TCs) showing differential hybridization signals in IL12-4 vs. M82. Click here for file

Additional file 4 **Ontology categorization for Cellular Component**. Functional categorization according to GO Cellular Component (CC) vocabulary of Tentative Consensus (TCs) showing differential hybridization signals in IL12-4 vs. M82. Click here for file

Additional file 5 **Heat map of upregulated transcripts**. The map was obtained from HCL clustering using Pearson's correlation as metrics. Normalized log transformed microarray data are represented according to a color scale. Click here for file

Additional file 6 **Heat map of downregulated transcripts**. The map was obtained from HCL clustering using Pearson's correlation as metrics. Normalized log transformed microarray data are represented according to a color scale. Click here for file
