# Supplementary material for: The ascorbic acid content of tomato fruits is associated with the expression of genes involved in pectin degradation
Source: BMC Plant Biol. 2010 Aug 6;10:163. doi: 10.1186/1471-2229-10-163 (PMC3095297; doi:10.1186/1471-2229-10-163)
Supplement: Additional file 1 — List of 253 probes showing differential hybridization signals in the IL12-4 and M82 transcriptomes. Statistical and annotation details of 253 probes showing differential hybridization signals at a 2-Factorial ANOVA model (P < 0.01) in the IL12-4 transcriptome compared to M82. Statistical analysis was performed by the TM4:MeV microarray software suite and the IL 12-4 vs. M82 signal ratio together with the Adjusted P value were reported. The automatic BLAST annotation of TC sequences was performed by the BLAST2GO software suite and the expectation value (e value), sequence similarity and corresponding Gene Ontology terms were reported. TCs are listed according to a decreasing IL12-4 vs. M82 fold change. [file 1471-2229-10-163-S1.DOC]

**Additional file 1**. Statistical and annotation details of 253 probes showing differential hybridization signals at a 2-Factorial ANOVA model (P<0.01) in the IL12-4 transcriptome compared to M82. Statistical analysis was performed by the TM4:MeV microarray software suite and the IL 12-4 *vs*. M82 signal ratio together with the Adjusted P value were reported. The automatic BLAST annotation of TC sequences was performed by the BLAST2GO software suite and the expectation value (e value), sequence similarity and corresponding Gene Ontology terms were reported. TCs are listed according to a decreasing IL12-4 *vs.* M82 fold change.

| **Sequence ID** | **Fold change** | **P value** | **Annotation** | | **Mean E value** | | | **Mean similarity** | |
| --- | --- | --- | --- | --- | --- | --- | --- | --- | --- |
| TC187915 | 6.601 | 1.00E-04 | NA | | - | | | - | |
| TC177433 | 6.403 | 7.84E-03 | tyramine hydroxycinnamoyltransferase | | 6.70E-127 | | | 71.20 | |
| TC172849 | 4.864 | 6.53E-06 | pyridoxal-phosphate dependent TrpB-like enzyme | | 2.16E-76 | | | 84.35 | |
| TC188751 | 4.842 | 1.39E-03 | NA | | 9.58E-11 | | | 85.00 | |
| TC177576 | 4.439 | 1.16E-03 | pectinesterase family protein | | 3.05E-92 | | | 60.05 | |
| TC180786 | 4.167 | 1.88E-03 | Wound-responsive protein | | 6.20E-139 | | | 78.20 | |
| TC172574 | 4.066 | 3.17E-03 | NA | | - | | | - | |
| TC183991 | 3.812 | 2.36E-03 | pyridoxal-phosphate-dependent TrpB-like enzyme | | 7.80E-142 | | | 80.70 | |
| TC170370 | 3.478 | 3.59E-05 | f-box family protein | | 7.06E-39 | | | 53.00 | |
| TC174199 | 3.175 | 9.02E-03 | seven transmembrane protein mlo8 | | 3.20E-133 | | | 77.50 | |
| TC188177 | 2.776 | 3.17E-03 | atp-binding sub-family b (mdr tap)member 10 | | 7.40E-140 | | | 74.30 | |
| TC184220 | 2.611 | 1.96E-03 | NA | | - | | | - | |
| TC184006 | 2.504 | 6.41E-03 | cystathionine gamma-synthase | | 6.40E-123 | | | 90.40 | |
| TC178718 | 2.495 | 6.91E-03 | protein | | 1.20E-110 | | | 73.80 | |
| TC182308 | 2.439 | 8.69E-03 | 21kd protein precursor | | 2.14E-21 | | | 93.85 | |
| TC182681 | 2.324 | 7.28E-03 | tryptophanyl-trna synthetase | | 1.31E-76 | | | 80.90 | |
| TC184472 | 2.276 | 7.67E-03 | NA | | - | | | - | |
| TC190989 | 2.269 | 1.65E-03 | NA | | - | | | - | |
| TC177185 | 2.234 | 2.73E-03 | propionyl-carboxylase beta chain | | 6.94E-71 | | | 78.60 | |
| TC190249 | 2.221 | 6.59E-03 | NA | | - | | | - | |
| TC185562 | 2.188 | 2.71E-03 | NA | | 2.59E-17 | | | 69.00 | |
| TC172667 | 2.180 | 6.50E-04 | pollen ole e 1 allergen and extensin family protein | | 2.81E-29 | | | 58.30 | |
| TC190538 | 2.168 | 7.37E-04 | NA | | - | | | - | |
| TC182898 | 2.167 | 3.64E-04 | dag protein | | 4.85E-27 | | | 78.88 | |
| TC179177 | 2.116 | 7.74E-03 | NA | | 1.19E-11 | | | 84.00 | |
| TC186434 | 2.073 | 3.94E-03 | NA | | - | | | - | |
| TC184744 | 2.073 | 6.06E-03 | NA | | - | | | - | |
| TC178115 | 2.001 | 6.02E-03 | NA | | 2.71E-26 | | | 55.50 | |
| TC176250 | 1.873 | 1.49E-05 | NA | | 1.3E-35 | | | 62.55 | |
| TC190107 | 1.867 | 7.76E-03 | phospho-glycerate kinase | | 6.48E-34 | | | 90.95 | |
| TC178728 | 1.779 | 2.03E-03 | ac006341_1ests gb | | 3.73E-23 | | | 78.50 | |
| TC180273 | 1.691 | 8.09E-03 | dna repair protein rad50 | | 4.70E-109 | | | 91.30 | |
| TC174528 | 1.646 | 7.57E-04 | anther-specific proline-rich protein apg | | 4.54E-88 | | | 66.50 | |
| TC177640 | 1.615 | 6.37E-03 | auxilin-like protein | | 1.56E-41 | | | 76.65 | |
| TC189398 | 1.567 | 2.68E-03 | ubiquitin-conjugating enzyme e2-17 kda | | 8.49E-32 | | | 98.20 | |
| TC182193 | 1.539 | 7.27E-03 | rubisco subunit binding-protein alpha subunit | | 8.34E-63 | | | 92.75 | |
| TC174575 | 1.532 | 2.15E-03 | proline-rich apg-like protein | | 2.16E-24 | | | 82.85 | |
| TC185095 | 1.522 | 9.99E-04 | membrane related | | 5.65E-79 | | | 65.30 | |
| TC180558 | 1.513 | 5.38E-03 | glycosyl hydrolase family 17 protein | | 4.82E-86 | | | 76.10 | |
| TC169916 | 1.444 | 7.26E-03 | 1-aminocyclopropane-1-carboxylate synthase | | 0.00 | | | 89.85 | |
| TC191252 | 1.429 | 4.28E-03 | NA | | - | | | - | |
| TC188390 | 1.428 | 5.56E-03 | NA | | 8.13E-14 | | | 73.00 | |
| TC185094 | 1.423 | 5.21E-03 | ribosomal protein s12 | | 7.35E-67 | | | 96.00 | |
| TC173750 | 1.411 | 3.02E-03 | NA | | 2.42E-15 | | | 61.00 | |
| TC181843 | 1.349 | 7.06E-03 | adp-ribosylation factor | | 2.86E-95 | | | 99.65 | |
| TC188955 | 1.305 | 3.19E-03 | carrier protein | | 4.14E-43 | | | 80.00 | |
| TC174366 | 1.192 | 5.75E-03 | chaperone protein dnaj-related-like | | 2.63E-89 | | | 81.30 | |
| TC186085 | 1.150 | 1.64E-03 | NA | | - | | | - | |
| TC178613 | 1.127 | 9.80E-03 | NA | | - | | | - | |
| TC189440 | 1.093 | 4.58E-03 | alpha tubulin | | 4.95E-39 | | | 99.30 | |
| TC173333 | 1.072 | 4.94E-03 | sulfate transporter | | 4.85E-51 | | | 70.85 | |
| TC181871 | 1.057 | 2.93E-03 | NA | | 8.88E-70 | | | 63.00 | |
| TC185020 | 1.011 | 4.43E-04 | nadp adrenodoxin-like ferredoxin reductase | | 1.37E-41 | | | 65.40 | |
| TC181936 | 1.009 | 8.90E-03 | at5g10910 t30n20_180 | | 5.26E-21 | | | 69.40 | |
| TC183220 | 0.961 | 7.29E-03 | pfkb-type carbohydrate kinase family protein | | 3.51E-18 | | | 78.75 | |
| TC184777 | 0.934 | 5.54E-03 | NA | | 1.47E-14 | | | 77.14 | |
| TC170812 | 0.863 | 5.05E-04 | amino acid permease-like protein | | 6.39E-79 | | | 84.35 | |
| TC178773 | 0.807 | 6.81E-03 | NA | | - | | | - | |
| TC187737 | 0.678 | 5.39E-03 | betaine aldehyde dehydrogenase-like | | 1.20E-104 | | | 78.70 | |
| TC190660 | 0.620 | 4.73E-03 | tubby protein | | 1.44E-32 | | | 73.35 | |
| TC175630 | 0.579 | 8.15E-03 | squalene synthase | | 5.20E-163 | | | 93.10 | |
| TC170080 | -0.500 | 7.33E-03 | aluminum-induced protein | | 1.10E-108 | | | 81.40 | |
| TC186336 | -0.537 | 2.79E-03 | amino acid transporter family protein | | 1.41E-72 | | | 76.55 | |
| TC175970 | -0.542 | 1.31E-03 | spermidine synthase | | 8.97E-91 | | | 86.40 | |
| TC170722 | -0.653 | 9.88E-03 | alpha-adr (alpha-adaptin) binding protein binding protein transporter | | 1.20E-116 | | | 76.45 | |
| TC182548 | -0.685 | 8.99E-03 | casein kinase ii subunit beta-4 | | 4.39E-63 | | | 80.95 | |
| TC186150 | -0.685 | 2.45E-03 | cytochrome p450 | | 3.45E-80 | | | 71.60 | |
| TC182124 | -0.687 | 8.54E-03 | peptidase s9 prolyl oligopeptidase active site domain protein | | 8.00E-168 | | | 73.10 | |
| TC180849 | -0.706 | 9.56E-03 | proteinase inhibitor i | | 4.43E-38 | | | 80.45 | |
| TC170477 | -0.732 | 7.16E-03 | imidazoleglycerol-phosphate dehydratase | | 1.78E-93 | | | 91.35 | |
| TC189505 | -0.747 | 6.97E-03 | protein kinase family protein | | 9.33E-30 | | | 74.95 | |
| TC186083 | -0.778 | 6.97E-03 | protein | | 2.30E-151 | | | 68.20 | |
| TC185832 | -0.781 | 5.01E-03 | mucin-like protein | | 2.50E-77 | | | 76.53 | |
| TC183109 | -0.783 | 9.23E-03 | NA | | - | | | - | |
| TC184213 | -0.791 | 2.59E-03 | sgt1-like protein | | 8.41E-92 | | | 78.85 | |
| TC177814 | -0.806 | 7.00E-03 | 20s rrnahomolog | | 1.12E-83 | | | 66.40 | |
| TC175356 | -0.821 | 2.84E-04 | niemann pick type c1 | | 2.90E-101 | | | 77.10 | |
| TC182304 | -0.836 | 9.62E-03 | rna binding motif protein 5 | | 2.00E-44 | | | 69.15 | |
| TC189481 | -0.839 | 2.23E-03 | NA | | - | | | - | |
| TC182168 | -0.844 | 6.38E-03 | ac006341_31 ests gb | | 1.47E-78 | | | 68.00 | |
| TC182279 | -0.857 | 7.99E-03 | NA | | 2.9E-24 | | | 56.67 | |
| TC184270 | -0.862 | 1.21E-04 | polyubiquitin | | 7.88E-69 | | | 99.65 | |
| TC177860 | -0.889 | 9.23E-03 | acyl-thioesterase ii | | 4.30E-113 | | | 73.80 | |
| TC178709 | -0.891 | 9.30E-03 | NA | | - | | | - | |
| TC180963 | -0.897 | 6.03E-03 | atpob1 (arabidopsis thaliana poz btb containing-protein 1) protein binding | | 1.06E-24 | | | 63.25 | |
| TC188118 | -0.897 | 5.44E-03 | protein kinase | | 0.00 | | | | 0.841 |
| TC188915 | -0.899 | 7.33E-03 | sec23 sec24 transport family protein | | 3.34E-22 | | | 80.90 | |
| TC172320 | -0.902 | 6.08E-03 | s-adenosylmethionine-dependent methyltransferase | | 7.15E-71 | | | 74.95 | |
| TC189173 | -0.910 | 1.02E-03 | emb1967 (embryo defective 1967) | | 7.74E-59 | | | 53.80 | |
| TC190353 | -0.913 | 6.91E-03 | kh domain-containing protein | | 6.03E-45 | | | 72.95 | |
| TC181908 | -0.924 | 4.02E-03 | NA | | - | | | - | |
| TC178320 | -0.930 | 6.01E-03 | NA | | 6.84E-60 | | | 54.86 | |
| TC189706 | -0.935 | 8.13E-03 | serine threonine protein kinase | | 3.10E-122 | | | 92.40 | |
| TC172295 | -0.949 | 9.07E-03 | structural molecule | | 4.53E-37 | | | 68.67 | |
| TC179858 | -0.949 | 6.53E-03 | rna-binding protein | | 7.10E-90 | | | 70.15 | |
| TC187309 | -0.955 | 7.49E-03 | vamp-associated protein | | 6.97E-27 | | | 70.55 | |
| TC172100 | -0.971 | 2.53E-03 | proline dehydrogenase | | 0.00 | | | 77.35 | |
| TC176303 | -0.983 | 3.40E-03 | rna-binding protein | | 2.29E-83 | | | 74.95 | |
| TC182440 | -0.991 | 4.44E-03 | UDP-3-O-[3-hydroxymyristoyl] N-acetylglucosamine deacetylase | | 1.10E-103 | | | 69.55 | |
| TC172922 | -1.000 | 9.92E-03 | pyrroline-5-carboxylate synthetase | | 0.00 | | | 0.8955 | |
| TC174673 | -1.005 | 9.64E-03 | nucleotide-sugar transporter family protein | | 8.89E-60 | | | 70.80 | |
| TC188650 | -1.008 | 6.05E-04 | atp-dependent metalloprotease-like protein | | 5.23E-92 | | | 73.30 | |
| TC187369 | -1.014 | 5.16E-03 | lrk1 protein | | 2.40E-70 | | | 54.95 | |
| TC177621 | -1.017 | 7.49E-03 | germin-like protein 4 | | 2.78E-87 | | | 82.70 | |
| TC180748 | -1.017 | 3.53E-04 | nucellin-like protein | | 6.43E-75 | | | 73.80 | |
| TC186510 | -1.026 | 9.65E-03 | heparanase-like protein 2expressed | | 6.65E-23 | | | 67.25 | |
| TC188063 | -1.035 | 7.67E-03 | subtilisin-like serine protease | | 1.27E-18 | | | 56.15 | |
| TC184729 | -1.038 | 4.24E-03 | transcriptional adaptor | | 2.32E-68 | | | 77.65 | |
| TC172099 | -1.044 | 7.42E-03 | calcium-binding ef hand family protein | | 0.00 | | | 0.8335 | |
| TC177614 | -1.044 | 9.79E-04 | nucleolar protein gar2-related | | 8.96E-34 | | | 67.35 | |
| TC185014 | -1.044 | 8.86E-03 | ankyrin repeat family protein | | 2.40E-50 | | | 77.56 | |
| TC173711 | -1.047 | 6.00E-04 | NA | | - | | | - | |
| TC176168 | -1.062 | 9.87E-03 | ammonium transporter | | 0.00 | | | 86.00 | |
| TC172234 | -1.065 | 5.55E-03 | NA | | - | | | - | |
| TC178473 | -1.071 | 4.23E-03 | fiber protein fb34 | | 8.51E-84 | | | 69.20 | |
| TC180289 | -1.071 | 6.15E-03 | NA | | - | | | - | |
| TC177242 | -1.077 | 8.19E-03 | ferric reductase-like transmembrane component | | 5.88E-33 | | | 66.54 | |
| TC176701 | -1.080 | 2.58E-05 | NA | | - | | | - | |
| TC181740 | -1.080 | 4.18E-03 | isovaleryl-dehydrogenase | | 1.00E-126 | | | 88.70 | |
| TC177160 | -1.083 | 3.98E-03 | tpa_inf: aro1-like protein 1 | | 1.16E-82 | | | 79.10 | |
| TC181406 | -1.095 | 1.84E-03 | cysteine synthase | | 3.30E-136 | | | 87.60 | |
| TC182492 | -1.095 | 5.69E-03 | na+ h+ antiporter | | 1.00E-178 | | | 83.25 | |
| TC173939 | -1.098 | 3.58E-04 | NA | | - | | | - | |
| TC173312 | -1.114 | 1.57E-03 | immunophilin | | 2.82E-61 | | | 82.70 | |
| TC182575 | -1.117 | 8.31E-03 | ribonuclease ii | | 1.60E-103 | | | 66.40 | |
| TC176620 | -1.123 | 1.54E-03 | ctp synthase like protein | | 8.58E-17 | | | 64.00 | |
| TC176752 | -1.123 | 4.95E-03 | beta-glycosyltransferase-like protein ii | | 6.70E-110 | | | 74.45 | |
| TC180107 | -1.123 | 7.29E-03 | y5390_arathame: full=uncharacterized protein at5g03900 flags: precursor | | 0.00 | | 0.715 | | |
| TC173053 | -1.129 | 5.20E-03 | mpt family transporter: inner membrane translocasetim44 | | 3.51E-73 | | | 62.58 | |
| TC183070 | -1.136 | 4.50E-03 | af439849_1 at2g31130 | | 1.56E-22 | | | 64.67 | |
| TC182589 | -1.152 | 4.40E-04 | dihydrofolate reductase-thymidylate synthase | | 0.00 | | | 83.40 | |
| TC172505 | -1.165 | 5.29E-03 | pyruvate kinase | | 9.10E-111 | | | 75.45 | |
| TC182248 | -1.198 | 2.23E-03 | beta-glucuronidase precursor | | 4.30E-166 | | | 66.80 | |
| TC188414 | -1.204 | 2.06E-03 | NA | | - | | | - | |
| TC172653 | -1.211 | 3.10E-03 | amplified in osteosarcoma | | 4.42E-85 | | | 63.50 | |
| TC171510 | -1.214 | 5.71E-03 | loc443610 protein | | 1.21E-98 | | | 81.80 | |
| TC186521 | -1.214 | 1.99E-04 | atpase-like protein | | 1.18E-49 | | | 78.65 | |
| TC172385 | -1.221 | 1.92E-03 | leucine-rich repeat family protein | | 7.60E-153 | | | 67.75 | |
| TC181855 | -1.224 | 2.53E-03 | at3g03330 t21p5_25 | | 1.61E-27 | | | 78.78 | |
| TC171305 | -1.231 | 7.64E-03 | protein | | 1.27E-46 | | | 68.33 | |
| TC186460 | -1.238 | 1.58E-04 | 24-dehydrocholesterol reductase | | 0.00 | | | 0.8235 | |
| TC182944 | -1.244 | 1.80E-03 | rna polymerase ii associated protein 3 | | 2.19E-53 | | | 68.80 | |
| TC185939 | -1.248 | 2.33E-03 | ubiquitin-protein ligase 1 | | 1.03E-50 | | | 93.20 | |
| TC188965 | -1.262 | 9.99E-03 | NA | | - | | | - | |
| TC188776 | -1.272 | 4.04E-03 | dof zinc finger protein | | 1.13E-33 | | | 79.10 | |
| TC173963 | -1.300 | 6.07E-03 | NA | | 2.25E-12 | | | 44.50 | |
| TC180123 | -1.322 | 9.36E-03 | mgc86284 protein | | 2.64E-84 | | | 70.75 | |
| TC172881 | -1.358 | 8.29E-03 | ascorbate peroxidase | | 2.73E-65 | | | 95.25 | |
| TC170324 | -1.370 | 5.80E-03 | beta-glucosidase 01 | | 8.10E-170 | | | 73.90 | |
| TC190625 | -1.381 | 8.26E-03 | homeodomain protein ahdp | | 3.50E-170 | | | 86.85 | |
| TC174342 | -1.385 | 2.22E-03 | selt-like protein precursor | | 7.22E-51 | | | 77.75 | |
| TC184848 | -1.411 | 3.59E-03 | cenp-e-like kinetochore protein | | 7.94E-37 | | | 62.50 | |
| TC185325 | -1.411 | 6.09E-03 | aspartyl protease family protein | | 4.40E-118 | | | 72.25 | |
| TC189544 | -1.423 | 7.97E-03 | 4-nitrophenylphosphatase-like protein | | 1.30E-15 | | | 79.10 | |
| TC175963 | -1.427 | 6.58E-03 | emb1923 (embryo defective 1923) | | 6.20E-93 | | | 75.00 | |
| TC178891 | -1.430 | 5.64E-03 | aspartate aminotransferase | | 1.32E-64 | | | 75.00 | |
| TC184514 | -1.430 | 9.44E-03 | atp binding domain 1member b | | 1.27E-77 | | | 85.90 | |
| TC187181 | -1.446 | 4.26E-05 | integral membrane protein | | 4.10E-161 | | | 84.30 | |
| TC182341 | -1.450 | 6.35E-03 | chromodomain helicase dna binding protein 1 | | 0.00 | | | 53.10 | |
| TC189332 | -1.454 | 9.99E-03 | at1g71940 f17m19_9 | | 1.76E-61 | | | 90.30 | |
| TC184166 | -1.458 | 5.15E-03 | proline iminopeptidase | | 4.80E-138 | | | 82.10 | |
| TC171546 | -1.462 | 7.17E-03 | potential gpi-protein transamidase complex subunit | | 1.30E-160 | | | 78.75 | |
| TC176012 | -1.486 | 6.33E-03 | peptide transporter | | 1.20E-112 | | | 85.10 | |
| TC188991 | -1.490 | 9.46E-03 | 60s ribosomal protein l5 | | 1.20E-123 | | | 90.95 | |
| TC177544 | -1.494 | 2.07E-03 | pollen surface protein | | 1.75E-48 | | | 80.70 | |
| TC188372 | -1.498 | 1.67E-03 | dual specificity protein phosphatase family protein | | 2.14E-85 | | | 74.60 | |
| TC184272 | -1.510 | 7.54E-04 | aldo keto reductase | | 8.80E-166 | | | 78.70 | |
| TC177420 | -1.515 | 2.08E-03 | NA | | 9.47E-18 | | | 79.00 | |
| TC187281 | -1.515 | 9.77E-03 | peroxisomal membrane 22 kda family protein | | 1.58E-74 | | | 62.83 | |
| TC173931 | -1.527 | 2.94E-03 | vacuolar sorting receptor | | 7.79E-18 | | | 83.64 | |
| TC180362 | -1.544 | 7.99E-03 | at5g66250 k1l20_3 | | 1.80E-131 | | | 74.67 | |
| TC174166 | -1.548 | 8.56E-03 | ring-h2 finger proteinexpressed | | 4.20E-69 | | | 73.05 | |
| TC183913 | -1.548 | 2.16E-03 | NA | | 4.29E-19 | | | 82.00 | |
| TC181437 | -1.552 | 5.86E-03 | ribosomal protein l21 | | 1.43E-41 | | | 76.75 | |
| TC175417 | -1.569 | 2.52E-03 | cleavage and polyadenylation specificity factor 5 | | 1.50E-104 | | | 87.85 | |
| TC173158 | -1.595 | 2.54E-03 | histone h3 | | 7.26E-59 | | | 98.40 | |
| TC174069 | -1.608 | 1.08E-04 | 3-hydroxyisobutyrate dehydrogenase | | 1.97E-67 | | | 69.85 | |
| TC172739 | -1.630 | 6.70E-03 | alanine racemase family protein | | 1.10E-103 | | | 82.95 | |
| TC187855 | -1.630 | 9.28E-03 | NA | | 6.24E-58 | | | 79.70 | |
| TC182904 | -1.644 | 6.35E-03 | elongation factor tu | | 0.00 | | | 0.8955 | |
| TC170307 | -1.653 | 8.90E-03 | mutant cincinnata | | 8.30E-154 | | | 63.20 | |
| TC171807 | -1.653 | 5.75E-03 | ubiquitin-conjugating enzyme e2-21 kda 1 | | 9.06E-95 | | | 89.45 | |
| TC180189 | -1.667 | 1.20E-03 | c9h6orf115 protein | | 4.33E-24 | | | 85.05 | |
| TC176540 | -1.676 | 1.30E-04 | protein | | 2.72E-61 | | | 92.55 | |
| TC181624 | -1.676 | 2.16E-03 | small nuclear ribonucleoprotein g | | 3.66E-34 | | | 93.05 | |
| TC175134 | -1.694 | 5.01E-04 | pde225 ptac7 (pigment defective 225) | | 2.07E-44 | | | 77.50 | |
| TC179231 | -1.699 | 4.73E-03 | protein | | 5.10E-111 | | | 73.80 | |
| TC178470 | -1.727 | 5.25E-03 | protein | | 3.00E-99 | | | 69.05 | |
| TC174061 | -1.742 | 4.63E-03 | fas protein | | 1.26E-64 | | | 74.90 | |
| TC179828 | -1.742 | 4.26E-03 | gamyb-binding protein | 0.00 | | | | 0.785 | |
| TC190833 | -1.796 | 6.11E-03 | minor allergen | | 7.14E-82 | | | 80.35 | |
| TC176350 | -1.832 | 2.09E-03 | 60s ribosomal protein l36 | | 4.61E-51 | | | 90.65 | |
| TC179680 | -1.884 | 2.18E-03 | 6,7-dimethyl-8-ribityllumazine synthase precursor expressed in Solanum chacosense | | 1.57E-88 | | | 77.65 | |
| TC175198 | -1.905 | 8.97E-03 | uncharacterised protein family containingexpressed | | 1.14E-50 | | | 71.50 | |
| TC188336 | -1.921 | 4.01E-03 | NA | | - | | | - | |
| TC189812 | -1.922 | 6.08E-03 | cyclopropyl isomerase | | 2.67E-85 | | | 78.17 | |
| TC180780 | -1.943 | 1.33E-04 | at5g40670 mnf13_190 | | 2.12E-52 | | | 76.40 | |
| TC179574 | -1.971 | 4.32E-03 | ubx domain-containing protein | | 6.95E-51 | | | 64.10 | |
| TC170315 | -1.994 | 1.48E-03 | pyrophosphate-dependent phosphofructo-1-kinase | | 8.30E-159 | | | 87.05 | |
| TC170849 | -2.059 | 1.95E-03 | at5g14910 f2g14_30 | | 2.42E-31 | | | 82.58 | |
| TC174858 | -2.071 | 2.91E-06 | xs domain containingexpressed | | 2.20E-116 | | | 60.80 | |
| TC181342 | -2.071 | 5.80E-03 | subtilisin-like protease | | | 0.00 | | 79.25 | |
| TC185313 | -2.120 | 6.34E-03 | transcription initiation factor | | 1.42E-61 | | | 71.93 | |
| TC180781 | -2.198 | 9.51E-05 | atp synthase subunit h family protein | | 8.96E-33 | | | 89.06 | |
| TC170700 | -2.232 | 5.62E-04 | seven transmembrane domain protein | | 1.50E-116 | | | 81.80 | |
| TC186276 | -2.272 | 1.89E-04 | dsba-like thioredoxin domain containing protein | | 1.61E-78 | | | 69.25 | |
| TC172075 | -2.293 | 9.89E-03 | synaptobrevin-like protein | | 7.31E-98 | | | 85.55 | |
| TC176194 | -2.308 | 6.29E-03 | chloroplast photosystem ii reaction center protein | | 1.62E-85 | | | 81.40 | |
| TC180762 | -2.329 | 2.07E-03 | elongation factor p | | 1.76E-85 | | | 82.35 | |
| TC187945 | -2.351 | 5.07E-03 | NA | | - | | | - | |
| TC175959 | -2.403 | 4.05E-03 | at3g45010 f14d17_80 | | 0.00 | | | 80.75 | |
| TC173500 | -2.427 | 6.60E-03 | ribosomal protein l18 | | 1.49E-60 | | | 80.15 | |
| TC174916 | -2.427 | 5.09E-06 | quinone oxidoreductase-like protein | | 7.90E-161 | | | 84.10 | |
| TC181458 | -2.427 | 3.10E-04 | homoaconitate hydratase family protein | | 9.73E-96 | | | 82.10 | |
| TC180224 | -2.442 | 6.75E-03 | monoterpene glucosyltransferase | | 0.00 | | | 71.30 | |
| TC183877 | -2.466 | 1.01E-03 | NA | | 6.78E-22 | | | 71.75 | |
| TC179410 | -2.482 | 1.36E-03 | at5g45690 mra19_8 | | 2.42E-97 | | | 72.65 | |
| TC186418 | -2.498 | 2.00E-04 | NA | | 1.61E-25 | | | 76.20 | |
| TC180251 | -2.582 | 4.78E-03 | at3g22430 mcb17_17 | | 2.44E-43 | | | 69.63 | |
| TC185917 | -2.617 | 7.51E-03 | 60s ribosomal protein l23 | | 7.59E-73 | | | 99.30 | |
| TC181418 | -2.635 | 2.18E-03 | histone h4 | | 1.19E-38 | | | 100.00 | |
| TC173927 | -2.689 | 7.38E-03 | 50s ribosomal protein l13 | | 2.46E-97 | | | 84.05 | |
| TC174973 | -2.718 | 2.90E-03 | nucleotide-binding protein | | 2.70E-107 | | | 91.55 | |
| TC171197 | -2.756 | 9.32E-03 | 26s proteasome non-atpase regulatory subunit 8 | | 2.50E-119 | | | 83.75 | |
| TC189571 | -2.756 | 5.75E-05 | NA | | - | | | - | |
| TC187316 | -2.776 | 6.24E-04 | ubiquitin-associated ts-n domain-containing protein | | 1.49E-65 | | | 74.30 | |
| TC176163 | -2.806 | 7.38E-03 | got1-like family protein | | 1.18E-23 | | | 74.25 | |
| TC175519 | -2.816 | 1.26E-03 | thioesterase family protein | | 6.19E-35 | | | 68.00 | |
| TC179109 | -2.943 | 5.18E-04 | protein | | 1.34E-54 | | | 77.92 | |
| TC170015 | -3.198 | 2.03E-03 | stress-related protein | | 4.77E-68 | | | 67.80 | |
| TC184801 | -3.308 | 1.36E-04 | alcohol dehydrogenase | | 3.25E-38 | | | 81.65 | |
| TC177541 | -3.336 | 8.83E-03 | NA | | 1.69E-31 | | | 83.40 | |
| TC177696 | -3.336 | 6.03E-03 | lipoic acid synthetase | | 2.59E-44 | | | 86.40 | |
| TC174250 | -3.381 | 9.64E-03 | fiber protein fb2 | | 4.38E-42 | | | 69.15 | |
| TC187614 | -3.411 | 8.37E-03 | ornithine carbamoyltransferase | | 1.82E-53 | | | 59.80 | |
| TC173094 | -3.442 | 4.03E-04 | ac012190_11 it is a member of an uncharacterised protein family pf | | 3.32E-75 | | | 86.55 | |
| TC176617 | -3.506 | 4.67E-03 | rpt2 (root phototropism 2) protein binding | | 8.79E-45 | | | 68.35 | |
| TC171334 | -3.573 | 1.56E-04 | 60s ribosomal proteinbbc1 protein | | 4.06E-90 | | | 91.55 | |
| TC176952 | -3.608 | 3.52E-04 | sec14 cytosolic factor family protein phosphoglyceride transfer family protein | | 9.10E-124 | | | 78.35 | |
| TC182497 | -3.776 | 2.16E-04 | calmodulin | | 2.73E-79 | | | 99.65 | |
| TC172446 | -4.012 | 1.06E-03 | aldo keto reductase | | 0.00 | | | 0.875 | |
| TC176849 | -4.035 | 2.24E-03 | 60s ribosomal protein l29 | | 3.45E-24 | | | 92.95 | |
| TC180230 | -4.265 | 2.18E-03 | pto kinase interactor 1 | | 0.00 | | | 89.25 | |
| TC180552 | -4.573 | 5.86E-03 | leucine-rich repeat receptor-like kinase | | 8.10E-89 | | | 55.05 | |
| TC181592 | -5.381 | 1.97E-05 | rub1 conjugating enzyme | | 2.03E-96 | | | 91.00 | |
| TC175115 | -5.878 | 3.68E-04 | ubiquitin-conjugating enzymej1 | | 1.10E-117 | | | 73.40 | |
| TC181167 | -6.644 | 8.71E-05 | ac022472_2pigpen protein from mus musculus gb | | 1.33E-63 | | | 69.20 | |
| TC191179 | -6.644 | 7.92E-05 | zinc finger protein | | 2.01E-24 | | | 78.75 | |
| TC189208 | -6.966 | 2.66E-05 | nucleotide-binding protein 1 | | 1.86E-42 | | | 83.15 | |
| TC174238 | -8.966 | 7.63E-08 | family protein | | 1.30E-129 | | | 81.80 | |
| TC181963 | -8.966 | 1.80E-03 | phloem-specific lectin | | 3.02E-61 | | | 60.85 | |
| TC189778 | -8.966 | 8.74E-03 | af243369_1glutathione s-transferase gst 14 | | 3.36E-29 | | | 73.10 | |
| TC173262 | -12.288 | 3.39E-04 | amp-binding protein | | 1.20E-143 | | | 82.65 | |
