# Supplementary material for: The ascorbic acid content of tomato fruits is associated with the expression of genes involved in pectin degradation
Source: BMC Plant Biol. 2010 Aug 6;10:163. doi: 10.1186/1471-2229-10-163 (PMC3095297; doi:10.1186/1471-2229-10-163)
Supplement: Additional file 2 — Ontology categorization for Biological Process. Functional categorization according to GO Biological Process (BP) vocabulary of Tentative Consensus (TCs) showing differential hybridization signals in IL12-4 vs. M82. [file 1471-2229-10-163-S2.DOC]

**Additional file 2**. Functional categorization of Tentative Consensus (TCs) showing differential hybridization signals in IL12-4 *vs*. M82. Categorization was performed according to GO Biological Process (BP) vocabulary as retrieved through Blast2GO Gene Ontology mapping. Differentially expressed TCs were categorized both as whole and split into up-regulated and down-regulated TCs.

| **BP categories** | **Differentially expressed TCs** | | **Up-regulated TCs** | | **Down-regulated TCs** | |
| --- | --- | --- | --- | --- | --- | --- |
|  | **N°** | **%^*^** | **N°** | **%^*^** | **N°** | **%^*^** |
| transport | 25 | 14.79 | 6 | 16.22 | 19 | 14.39 |
| cellular component organization and biogenesis | 20 | 11.83 | 5 | 13.51 | 15 | 11.36 |
| amino acid and derivative metabolic process | 17 | 10.06 | 6 | 16.22 | 11 | 8.33 |
| protein modification process | 13 | 7.69 | 1 | 2.70 | 12 | 9.09 |
| translation | 12 | 7.10 | 2 | 5.41 | 10 | 7.58 |
| response to stress | 10 | 5.92 | 0 | - | 10 | 7.58 |
| multicellular organism development | 10 | 5.92 | 3 | 8.11 | 7 | 5.30 |
| catabolic process | 9 | 5.33 | 2 | 5.41 | 7 | 5.30 |
| lipid metabolic process | 8 | 4.73 | 3 | 8.11 | 5 | 3.79 |
| transcription | 8 | 4.73 | 2 | 5.41 | 6 | 4.55 |
| response to abiotic stimulus | 6 | 3.55 | 0 | - | 6 | 4.55 |
| carbohydrate metabolic process | 6 | 3.55 | 3 | 8.11 | 3 | 2.27 |
| signal transduction | 5 | 2.96 | 1 | 2.70 | 4 | 3.03 |
| response to extracellular stimulus | 4 | 2.37 | 0 | - | 4 | 3.03 |
| electron transport | 3 | 1.78 | 0 | - | 3 | 2.27 |
| response to endogenous stimulus | 3 | 1.78 | 0 | - | 3 | 2.27 |
| generation of precursor, metabolites and energy | 3 | 1.78 | 1 | 2.70 | 2 | 1.52 |
| secondary metabolic process | 3 | 1.78 | 2 | 5.41 | 1 | 0.76 |
| flower development | 2 | 1.18 | 0 | - | 2 | 1.52 |
| cell differentiation | 2 | 1.18 | 0 | - | 2 | 1.52 |
|  |  |  |  |  |  |  |
| total sequence with GO | 169 |  | 37 |  | 132 |  |
| unknown | 84 |  | 24 |  | 60 |  |
| total number of TCs | 253 |  | 61 |  | 192 |  |

* Calculated as percentage of the total number of classifications
