# Supplementary material for: The ascorbic acid content of tomato fruits is associated with the expression of genes involved in pectin degradation
Source: BMC Plant Biol. 2010 Aug 6;10:163. doi: 10.1186/1471-2229-10-163 (PMC3095297; doi:10.1186/1471-2229-10-163)
Supplement: Additional file 3 — Ontology categorization for Molecular Function. Functional categorization according to GO Molecular Function (MF) vocabulary of Tentative Consensus (TCs) showing differential hybridization signals in IL12-4 vs. M82. [file 1471-2229-10-163-S3.DOC]

**Additional file 3**. Functional categorization of Tentative Consensus (TCs) showing differential hybridization signals in IL12-4 *vs*. M82. Categorization was performed according to GO Molecular Function (MF) vocabulary as retrieved through Blast2GO Gene Ontology mapping. Differentially expressed TCs were categorized both as whole and split into up-regulated and down-regulated TCs.

| **MF categories** | **Differentially expressed TCs** | | **Up-regulated TCs** | | **Down-regulated TCs** | |
| --- | --- | --- | --- | --- | --- | --- |
|  | **N°** | **%^*^** | **N°** | **%^*^** | **N°** | **%^*^** |
| hydrolase activity | 23 | 21.10 | 8 | 32.00 | 15 | 17.86 |
| protein binding | 20 | 18.35 | 3 | 12.00 | 17 | 20.24 |
| nucleotide binding | 16 | 14.68 | 5 | 20.00 | 11 | 13.10 |
| structural molecule activity | 12 | 11.01 | 2 | 8.00 | 10 | 11.90 |
| kinase activity | 11 | 10.09 | 2 | 8.00 | 9 | 10.71 |
| transporter activity | 10 | 9.17 | 2 | 8.00 | 8 | 9.52 |
| RNA binding | 8 | 7.34 | 0 | - | 8 | 9.52 |
| enzyme regulator activity | 4 | 3.67 | 2 | 8.00 | 2 | 2.38 |
| transcription factor activity | 3 | 2.75 | 1 | 4.00 | 2 | 2.38 |
| receptor activity | 2 | 1.83 | 0 | - | 2 | 2.38 |
|  |  |  |  |  |  |  |
| total sequence with GO | 109 |  | 25 |  | 84 |  |
| unknown | 144 |  | 36 |  | 108 |  |
| total number of TCs | 253 |  | 61 |  | 192 |  |

* Calculated as percentage of the total number of classifications.
