# Supplementary material for: The ascorbic acid content of tomato fruits is associated with the expression of genes involved in pectin degradation
Source: BMC Plant Biol. 2010 Aug 6;10:163. doi: 10.1186/1471-2229-10-163 (PMC3095297; doi:10.1186/1471-2229-10-163)
Supplement: Additional file 4 — Ontology categorization for Cellular Component. Functional categorization according to GO Cellular Component (CC) vocabulary of Tentative Consensus (TCs) showing differential hybridization signals in IL12-4 vs. M82. [file 1471-2229-10-163-S4.DOC]

**Additional file 4.** Functional categorization of Tentative Consensus (TCs) showing differential hybridization signals in the IL12-4 *vs.* M82. Categorization was performed according to GO Cellular Component (CC) vocabulary as retrieved through Blast2GO Gene Ontology mapping. Differentially expressed TCs were categorized both as whole and stratified in up-regulated and down-regulated TCs.

| **CC categories** | **Differentially expressed TCs** | | **Up-regulated TCs** | | **Down-regulated TCs** | |
| --- | --- | --- | --- | --- | --- | --- |
|  | **N°** | **%^*^** | **N°** | **%^*^** | **N°** | **%^*^** |
| plastid | 39 | 33.91 | 10 | 40.00 | 29 | 32.22 |
| mitochondrion | 26 | 22.61 | 7 | 28.00 | 19 | 21.11 |
| ribosome | 10 | 8.70 | 1 | 4.00 | 9 | 10.00 |
| endoplasmic reticulum | 9 | 7.83 | 1 | 4.00 | 8 | 8.89 |
| plasma membrane | 8 | 6.96 | 1 | 4.00 | 7 | 7.78 |
| Golgi apparatus | 5 | 4.35 | 1 | 4.00 | 4 | 4.44 |
| nucleoplasm | 4 | 3.48 | 1 | 4.00 | 3 | 3.33 |
| cytosol | 4 | 3.48 | 1 | 4.00 | 3 | 3.33 |
| peroxisome | 2 | 1.74 | 0 | - | 2 | 2.22 |
| cytoskeleton | 2 | 1.74 | 2 | 8.00 | 0 | - |
| extracellular space | 2 | 1.74 | 0 | - | 2 | 2.22 |
| thylacoid | 2 | 1.74 | 0 | - | 2 | 2.22 |
| vacuole | 2 | 1.74 | 0 | - | 2 | 2.22 |
|  |  |  |  |  |  |  |
| total sequence with GO | 115 |  | 25 |  | 90 |  |
| unknown | 138 |  | 36 |  | 102 |  |
| total number of TCs | 253 |  | 61 |  | 192 |  |

* Calculated as percentage of the total number of classifications.
