# Supplementary figures and images for: The ascorbic acid content of tomato fruits is associated with the expression of genes involved in pectin degradation
Source: BMC Plant Biol. 2010 Aug 6;10:163. doi: 10.1186/1471-2229-10-163 (PMC3095297; doi:10.1186/1471-2229-10-163)

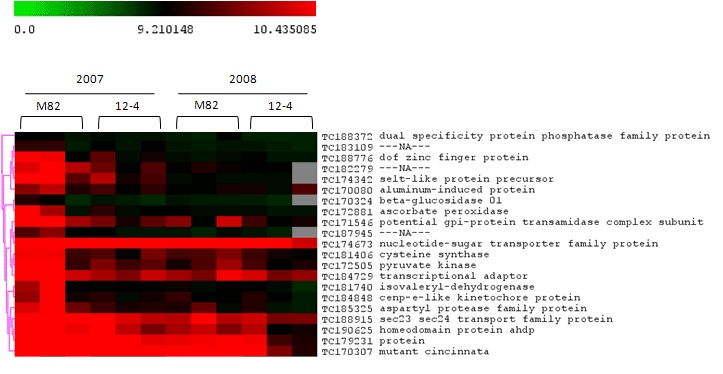

Supplement: Additional file 6 — Heat map of downregulated transcripts. The map was obtained from HCL clustering using Pearson's correlation as metrics. Normalized log transformed microarray data are represented according to a color scale. [file 1471-2229-10-163-S6.JPEG]
